# Endothelial cell-specific roles for tetrahydrobiopterin in myocardial function, cardiac hypertrophy, and response to myocardial ischemia-reperfusion injury

**DOI:** 10.1152/ajpheart.00562.2022

**Published:** 2023-02-03

**Authors:** Surawee Chuaiphichai, Sandy M. Chu, Ricardo Carnicer, Matthew Kelly, Jenifer K. Bendall, Jillian N. Simon, Gillian Douglas, Mark J. Crabtree, Barbara Casadei, Keith M. Channon

**Affiliations:** ^1^Division of Cardiovascular Medicine, British Heart Foundation Centre of Research Excellence, Radcliffe Department of Medicine, University of Oxford, Oxford, United Kingdom; ^2^Department of Biochemical Sciences, School of Bioscience and Medicine, University of Surrey, Guildford, United Kingdom; ^3^Center for Translational Medicine, Lewis Katz School of Medicine, Temple University, Philadelphia, Pennsylvania, United States

**Keywords:** cardiac function, hypertrophy, ischemia-reperfusion injury, nitric oxide synthase, tetrahydrobiopterin

## Abstract

The cofactor tetrahydrobiopterin (BH_4_) is a critical regulator of nitric oxide synthase (NOS) function and redox signaling, with reduced BH_4_ implicated in multiple cardiovascular disease states. In the myocardium, augmentation of BH_4_ levels can impact on cardiomyocyte function, preventing hypertrophy and heart failure. However, the specific role of endothelial cell BH_4_ biosynthesis in the coronary circulation and its role in cardiac function and the response to ischemia has yet to be elucidated. Endothelial cell-specific *Gch1* knockout mice were generated by crossing *Gch1^fl/fl^* with Tie2cre mice, generating *Gch1^fl/fl^*Tie2cre mice and littermate controls. GTP cyclohydrolase protein and BH_4_ levels were reduced in heart tissues from *Gch1^fl/fl^*Tie2cre mice, localized to endothelial cells, with normal cardiomyocyte BH_4_. Deficiency in coronary endothelial cell BH_4_ led to NOS uncoupling, decreased NO bioactivity, and increased superoxide and hydrogen peroxide productions in the hearts of *Gch1^fl/fl^*Tie2cre mice. Under physiological conditions, loss of endothelial cell-specific BH_4_ led to mild cardiac hypertrophy in *Gch1^fl/fl^*Tie2cre hearts. Endothelial cell BH_4_ loss was also associated with increased neuronal NOS protein, loss of endothelial NOS protein, and increased phospholamban phosphorylation at serine-17 in cardiomyocytes. Loss of cardiac endothelial cell BH_4_ led to coronary vascular dysfunction, reduced functional recovery, and increased myocardial infarct size following ischemia-reperfusion injury. Taken together, these studies reveal a specific role for endothelial cell *Gch1*/BH_4_ biosynthesis in cardiac function and the response to cardiac ischemia-reperfusion injury. Targeting endothelial cell *Gch1* and BH_4_ biosynthesis may provide a novel therapeutic target for the prevention and treatment of cardiac dysfunction and ischemia-reperfusion injury.

**NEW & NOTEWORTHY** We demonstrate a critical role for endothelial cell *Gch1*/BH_4_ biosynthesis in coronary vascular function and cardiac function. Loss of cardiac endothelial cell BH_4_ leads to coronary vascular dysfunction, reduced functional recovery, and increased myocardial infarct size following ischemia/reperfusion injury. Targeting endothelial cell *Gch1* and BH_4_ biosynthesis may provide a novel therapeutic target for the prevention and treatment of cardiac dysfunction, ischemia injury, and heart failure.

## Graphical abstract

**Figure:**
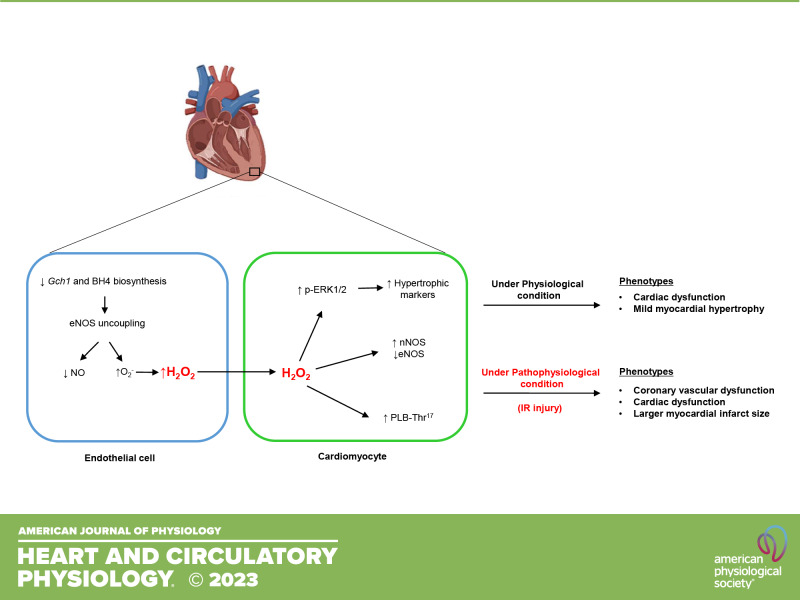

## INTRODUCTION

Cardiovascular diseases including coronary artery disease, myocardial infarction, and heart failure are leading causes of global mortality and disability (1). A hallmark of cardiovascular diseases is an early reduction in nitric oxide (NO) bioavailability and an increase in reactive oxygen species (ROS) production. Tetrahydrobiopterin (BH_4_) is a critical regulator of nitric oxide synthases (NOS) function and NOS-derived NO and ROS signaling in cardiovascular physiology (2, 3). Biosynthesis of BH_4_ is catalyzed by GTPCH (GTP cyclohydrolase 1, encoded by *Gch1*). We have previously shown that *Gch1* expression is a key determinant of BH_4_ bioavailability, NOS regulation, and NO generation (4, 5). When BH_4_ bioavailability is limited, NOS is unable to generate NO from l-arginine and becomes “uncoupled,” resulting in generation of superoxide anion and other ROS, rather than NO, contributing to disturbed redox signaling (3, 6–8).

Clinically, genetic variants in *GCH1* functionally associated with altered *GCH1* expression appear to be associated with alterations in markers of cardiac function and cardiovascular risk (9, 10). Rare genetic variants causing loss of BH_4_ synthesis are also associated with alterations of NOS-mediated vascular function and cardiovascular physiology (9, 10). These observations from clinical studies have been supported by preclinical models. Reduced BH_4_ bioavailability and NOS uncoupling are associated with various heart diseases including cardiac hypertrophy (11) and ischemia-reperfusion injury (12–17). Oral supplementation with BH_4_ or the BH_4_ precursor sepiapterin has been shown to prevent or reduce cardiac hypertrophy and failure (11, 18–21). For example, pressure overload induced by transverse aortic constriction (TAC) in mice reduced cardiac BH_4_ levels and promoted eNOS uncoupling, leading to myocyte hypertrophy, cardiac dilation, interstitial fibrosis, and ventricular dysfunction (22). Treatment with oral BH_4_ prevented the NOS uncoupling and reduced the TAC-induced hypertrophy. Furthermore, exogenous BH_4_ was able to recouple eNOS and reverse preexisting cardiac hypertrophy and fibrosis caused by TAC-induced pressure overload (11).

However, these and other studies do not address the important question of which cell types in the heart may mediate the effects of BH_4_ on cardiac function. NO is generated from coronary endothelial cells by eNOS, whereas in cardiac myocytes, both eNOS and nNOS have been shown to contribute to myocardial function. In the endothelium, NO mediates coronary vascular function and flow, whereas in cardiac myocytes, NO regulates LV relaxation by effects on myofilament calcium sensitivity and calcium handling (23). Although prior studies have suggested a critical role of endothelial NO and BH_4_ levels on cardiac function and injury (17, 21, 24–26), no prior study has specifically addressed the requirement for endothelial cell BH_4_ in the regulation of cardiac function, particularly in myocardial ischemia-reperfusion (I/R) injury where roles for both eNOS and nNOS are implicated (27). Interpreting the specific roles of NO in the heart using knockouts of either eNOS or nNOS is limited by loss of all NOS-related functions, including ROS generation, subcellular localization, and protein-protein interactions, whereas the requirement for BH_4_ in the generation of NO by both eNOS and nNOS enables selective targeting of NOS-mediated NO generation, without primary alterations in either NOS protein levels or other NOS functions.

Accordingly, we sought to investigate how selective targeting of endothelial cell BH_4_ biosynthesis, without alteration of cardiac myocyte BH_4_, would alter cardiac function, focusing on ischemia-reperfusion injury, where changes in coronary endothelial function play an important pathophysiological role.

## METHODS

### Animals

All animal studies were conducted under project licenses PPL 30/3080 and P0C27F69A with ethical approval from the Local Ethical Review Committee and in accordance with the United Kingdom Home Office regulations (Guidance on the Operation of Animals, Scientific Procedures Act), 1986, with procedures reviewed by the clinical medicine Animal Care and Ethical Review Body (AWERB). Animals were housed in individually ventilated cages (between 4 and 6 mice per cage of mixed genotypes) in specific pathogen-free conditions. All animals were provided with standard chow (Teklad global 16% protein diet, Harlan Laboratories) and water ad libitum and maintained on a 12-h:12-h light/dark cycle at controlled temperature (20–22°C) and humidity.

### *Gch1* Knockout Mice

We generated mice with a *Gch1* conditional knockout (floxed) allele, as previously described (28–30). *Gch1^fl/fl^* animals were bred with Tie2cre transgenic mice to produce *Gch1^fl/fl^*Tie2cre mice where *Gch1* is deleted in endothelial cells, generating a mouse model of endothelial cell-specific BH_4_ deficiency. Since the Tie2cre transgene is active in the female germline, only male animals are used to establish breeding pairs to maintain conditional endothelial cell expression. Mice were genotyped according to the published protocol (29, 31). Briefly, mice were genotyped by polymerase chain reactions using DNA prepared from ear biopsies. For *Gch1^fl/fl^* genotyping, PCR was performed using the following primers: Gch1*^fl/fl^*, forward 5′-GTC CTT GGT CTC AGT AAA CTT GCC AGG-3′; Gch1*^fl/fl^*, reverse 5′-GCC CAG CCA AGG ATA GAT GCA G-3′. The *Gch1* floxed allele showed as 1,030 bp. For Tie2cre genotyping, PCR was performed using the following primers: Tie2cre, forward 5′-GCA TAA CCA GTG AAA CAG CAT TGC TG-3′; Tie2cre, reverse 5′-GGA CAT GTT CAG GGA TCG CCA GGC G-3′. The Tie2cre allele amplified as 280-bp fragment. Adult male *Gch1^fl/fl^*Tie2cre mice and their *Gch1^fl/fl^* littermates (hereafter referred to as wild type) on a pure (>10 generations) C57BL6/J background were bred in house and were used for all experiments at 20 to 24 wk.

### Determination of Tissue Tetrahydrobiopterin Levels

BH_4_ and oxidized biopterins (BH_2_ and biopterin) were determined by high-performance liquid chromatography (HPLC) followed by electrochemical and fluorescence detection, respectively, following an established protocol (32). Briefly, frozen heart samples were homogenized in ice-cold resuspension buffer, consisting of (in mmol · L^−1^ ) 50 phosphate-buffered saline, 1 dithioerythriol, and 1 EDTA at pH 7.4. After centrifugation at 13,200 rpm for 10 min at 4°C, the supernatant was removed, and ice-cold acid precipitation buffer, consisting of (in mmol · L^−1^) 1 phosphoric acid, 2 trichloroacetic acid, and 1 dithioerythritol, was added. Samples were vigorously mixed and then centrifuged for 15 min at 13,000 rpm and 4°C. Samples were injected into an isocratic HPLC system and quantified using sequential electrochemical (Coulochem III, ESA, Inc.) and fluorescence (Jasco) detection. HPLC separation was performed using a 250-mm ACE C-18 column (Hichrom) and a mobile phase comprised of 50 mM sodium acetate, 5 mM citric acid, 48 µΜ EDTA, and 160 µΜ dithioerythritol (pH 5.2) (all ultrapure electrochemical HPLC grade) at a flow rate of 1.3 mL/min. Background currents of +500 μA and −50 μA were used for the detection of BH_4_ on electrochemical cells E1 and E2, respectively. 7,8-BH_2_ and biopterin were measured using a Jasco FP2020 fluorescence detector set at 510-nm excitation and 595-nm emission. Quantification of BH_4_, BH_2_, and B was done by comparison with authentic external standards and normalized to sample protein content.

### Endothelial Cell Isolation

Primary heart endothelial cells were isolated using MACS beads (Miltenyi Biotec), as previously described (29). Briefly, mice were euthanized by an overdose of inhaled isoflurane. Hearts were harvested and digested in DMEM containing 0.18 U/mL Liberase (Roche) and 0.1 mg/mL DnaseI (Roche) for 1 h at 37°C. The digested tissue was filtered through 100- and 70-μm cell strainers. The cell suspension was then incubated with rat anti-CD31 antibody (BD PharMingen) for 15 min at 4°C and then with anti-rat secondary antibody coated immune magnetic beads for a further 15 min at 4°C. Bead-bound endothelial cells were selected using a magnetic column. Endothelial cells were collected and stored at −80°C for further analysis.

### Cardiomyocyte Isolation

Cardiac myocytes were isolated using an enzymatic dispersion technique (23). Briefly, the heart was perfused with Ca^2+^-free isolation solution (37°C, oxygenated) for 3 min and then with 1 mg/mL collagenase type II solution (Worthington Biochemical) for a further 9 min. The myocytes were pelleted by centrifugation at a low speed (600 rpm). The supernatant was then spun down at 10,000 rpm; the pellet was considered as the nonmyocyte fraction.

### Echocardiography

Left ventricular (LV) size and function were investigated in vivo using a high-resolution two-dimensional (2D) echocardiography system (Vevo 2100, VisualSonics, Canada) in isoflurane (1%–1.5%) anesthetized mice. LV wall thickness and chamber dimensions were determined in the parasternal short-axis view (M-mode), from which measures of LVEF and fractional shortening were derived. 2-D images of the heart were obtained from the four-chamber apical view to assess mitral blood inflow and tissue-Doppler velocities.

### Quantification of Superoxide Production by Dihydroethidine (DHE)-HPLC

Superoxide production was quantified by measuring the production of 2-hydroxyethidium from dihydroethidium, using HPLC (29). Briefly, frozen heart homogenate was preincubated with serum-free DMEM with or without 100 μM *N*^G^-nitro-l-arginine methyl ester (l-NAME; Sigma). Samples were then incubated with 25 μM DHE (Invitrogen) for 20 min before being harvested for separation of 2-hydroxyethidium using a gradient HPLC system (Jasco, UK) with an ODS3 reverse phase column (250 mm, 4.5 mm, Hichrom UK) and quantified using a fluorescence detector set at 510 nm (excitation) and 595 nm (emission).

### Langendorff Heart Preparation

Mice were heparinized (300 U) and anesthetized with ketamine (75 mg/kg) plus medetomidine hydrochloride (1 mg/kg), with the adequacy of anesthesia confirmed by the absence of a pedal reflex. Hearts were quickly excised and immersed in KH buffer. The aorta was then cannulated onto the Langendorff perfusion system for retrograde perfusion. The heart was perfused with 37°C KH buffer and gassed with 95% O_2_-5% CO_2_, at 2 mL/min, and cardiac function was assessed using a fluid-filled balloon inserted into the left ventricle, which connected to a pressure transducer and a PowerLab system (ADInstruments). Left ventricular developed pressure (LVDP), calculated by the difference between systolic and diastolic pressure, was recorded continuously via LabChart software v.7.0. After 25-min equilibration, hearts were subjected to 35 min global ischemia followed by 60 min of reperfusion. For triphenyltetrazolium chloride (TTC) staining, hearts were removed from the Langendorff following ex vivo I/R, briefly frozen, and then sliced into six 1-mm-thick transverse sections. To distinguish viable (stained) versus necrotic (pale, unstained) tissue, sections were incubated in 1% TTC for 30 min at 37°C. Sections were then scanned, and the area of infarction (TTC negative) was quantified as a percentage of the area at risk (entire area of the section) using ImageJ.

### Quantification of Gene Expression by Real-Time RT-PCR

RNA was prepared using the RNeasy kit (Qiagen) and was reverse transcribed using Superscript II (Life Technologies) according to standard protocols. RNA equivalent cDNA (5 ng) was used to perform real-time PCR using predesigned tag-man gene expression assays (Life Technologies) using a BioRad CFX1000. Gene expression levels of mouse *Gch1*, *Nos1*, *Nos2*, and *Nos3* were normalized to the housekeeping gene *GAPDH* using the ΔCt method.

### Western Blot Analysis

Immunoblotting in LV homogenates was performed to evaluate protein levels of GTPCH (1:10,000 dilution; a gift from S.Gross, Cornell University; New York), iNOS (1: 1,100 dilution; Abcam), nNOS (1:1,000 dilution; Santa Cruz Biotechnology), eNOS (1:5,000 dilution; BD Bioscience), CD102 (1:1,000; R&D systems), SERCA2A (1:5,000 dilution; Santa Cruz Biotechnology), total phospholamban (1:2,000 dilution; PLB, Badrilla), phosphor-Thr17-PLB (1:2,000 dilution; Badrilla), phosphor-Ser16-PLB (1:2,000 dilution; Badrilla), NCX1 (1:1,000 dilution, Santa Cruz), phospho-extracellular signal-regulated protein kinases (1:500 dilution; ERK1/2), total ERK1/2 (1:500 dilution;), catalase (1:5,000 dilution; Calbiochem), MnSOD (1:5,000 dilution; Stressgen Bioreagents), EcSOD (1:750 dilution; Stressgen Bioreagents), Cu/ZnSOD (1:500 dilution; Stressgen Bioreagents), and β-tubulin (1:20,000; Abcam), followed by appropriate HRP-conjugated secondary antibody (1:10,000–20,000 dilution; Promega). Protein bands were visualized by enhanced chemiluminescence (Super West Pico Chemiluminescence, Thermo Scientific).

### Blood Pressure Measurement by Tail-Cuff Plethysmography

Systolic blood pressure in conscious wild-type and *Gch1^fl/fl^*Tie2cre mice was determined using the VisitechR computerized tail-cuff plethysmography system (Visitech) following 5 days of training and 3 days baseline periods. Experiments were performed between the hours of 8:00 and 12:00 am. The animal tails were passed through a cylindrical latex tail-cuff and taped down to reduce movement. Twenty readings were taken per mouse of which the first five readings were discarded. The remaining 15 readings were used to calculate the mean systolic blood pressure in each mouse.

### Statistical Analysis

All data are reported as means ± SE. The experimental unit (*n*) was defined as a single animal, animals of both genotypes were caged together, and animals of both genotypes were derived from more than one cage in all experiments. Statistical analyses were performed using GraphPad Prism v. 9.3.0. (San Diego, CA). Normality was tested using D’Agostino and Pearson omnibus normality test. Groups were compared using the Mann–Whitney *U* test for nonparametric data or an unpaired Student’s *t* test for parametric data. When comparing multiple groups, data were analyzed by analysis of variance (ANOVA) with Newman–Keuls posttest for parametric data or Kruskal–Wallis test with Dunn’s posttest for nonparametric data. When more than two independent variables were present, a two-way ANOVA with Tukey’s multiple comparisons test was used. When within-subject repeated measurements were present, a repeated-measures (RM) ANOVA was used. A value of *P* < 0.05 was considered statistically significant. Data were collected and analyzed with the operator blind of treatment allocation. Randomization was performed by cage.

## RESULTS

### Endothelial Cell-Targeted *Gch1* Deletion in the Heart Causes Selective Endothelial Cell BH_4_ Deficiency

**Figure 1.**
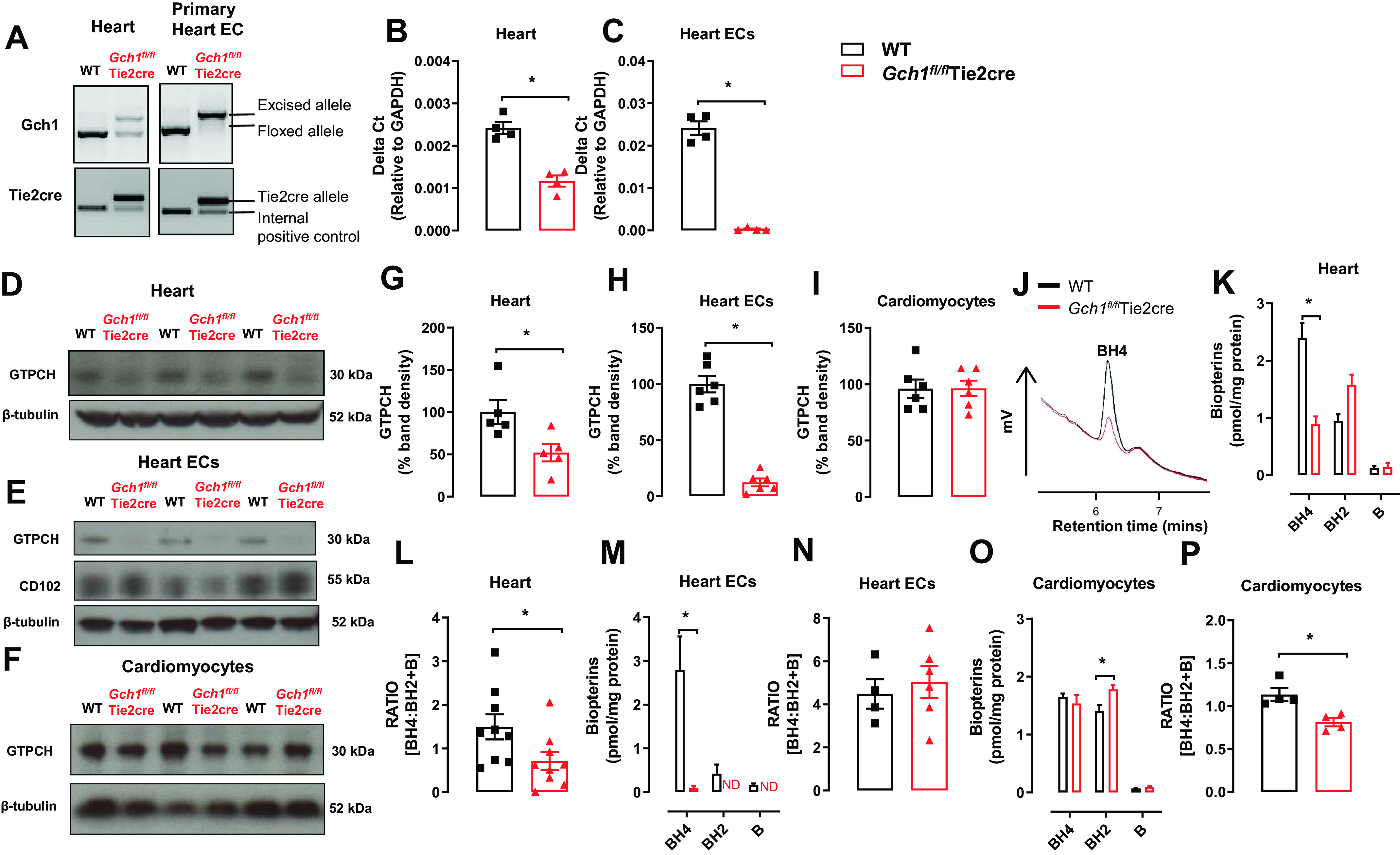
Myocardial endothelial cell targeted *Gch1* deletion causes a tissue-specific decrease in *Gch1* gene, GTPCH protein, and biopterin content. *A*: evaluation of Tie2cre-mediated excision of the loxP flanked DNA in heart tissues and primary heart endothelial cells derived from *Gch1^fl/fl^*Tie2cre and *Gch1^fl/fl^* [wild-type mice (WT)]. The predicted 1,030-bp product was detected in WT mice. In the presence of Tie2cre transgene a 1,392-bp knockout allele was detected, with efficient excision in primary endothelial cells from hearts. *B* and *C*: quantitative real-time PCR was used to quantify *Gch1* gene expression in hearts and primary endothelial cells from hearts (*P < 0.05; *n* = 4 per group). *D–F*: representative immunoblot of GTPCH proteins in hearts, isolated primary endothelial cells, and isolated cardiomyocytes from WT and *Gch1^fl/fl^*Tie2cre hearts, respectively, with quantitative data, measured as percent band density in *G–I*: CD102 and β-tubulin were used as endothelial cell marker and loading control respectively. *J*: representative chromatograms of BH_4_ traces in hearts from WT and *Gch1^fl/fl^*Tie2cre mice. *K* and *L*: BH_4_ levels and BH_4_/BH_2_ + B ratio were reduced in hearts from *Gch1^fl/fl^*Tie2cre mice compared with wild-type littermates (*P < 0.05; *n* = 8 and 9 per group). *M* and *N*: BH_4_ levels were barely detectable in primary ECs from *Gch1^fl/fl^*Tie2cre compared with WT mice (*P < 0.05; *n* = 4–6 per group). *O* and *P*: BH_4_ levels were comparable between primary cardiomyocytes from *Gch1^fl/fl^*Tie2cre mice and wild-type littermates. Absolute BH_2_ levels in cardiomyocytes were significantly increased in *Gch1^fl/fl^*Tie2cre mice compared with wild-type mice, such that the BH_4_/BH_2_ and biopterin ratio was significantly reduced in cardiomyocytes in *Gch1^fl/fl^*Tie2cre mice (*P < 0.05; *n* = 4 per group). Each data point represents an individual adult male mouse.

We generated matched litters of *Gch1^fl/fl^*Tie2cre and *Gch1^fl/fl^* mice (hereafter referred to as wild type) by crossing male *Gch1^fl/fl^*Tie2cre and female *Gch1^fl/fl^* mice. Body weights between the groups were similar (36 ± 1.5 g in wild type and 36 ± 1.1 g in *Gch1^fl/fl^*Tie2cre; *n* = 6 to 8 animals per group). Genomic polymerase chain reaction demonstrated efficient excision of the floxed *Gch1* allele in isolated endothelial cells from *Gch1^fl/fl^*Tie2cre hearts (Fig. 1*A*). Endothelial cell-specific *Gch1* deletion resulted in a significant reduction in *Gch1* expression (Fig. 1, *B* and *C*), GTPCH protein in whole heart tissue, and barely detectable levels in endothelial cells isolated from the hearts. However, the GTPCH protein in isolated cardiomyocytes was similar between the groups (Fig. 1, *D*–*I*). Accordingly, BH_4_ levels were significantly decreased in hearts and barely detected in isolated endothelial cells from *Gch1^fl/fl^*Tie2cre hearts (Fig. 1, *J*, *K*, and *M*). Despite marked BH_4_ deficiency, absolute BH_2_ levels in heart tissue were comparable between wild-type and *Gch1^fl/fl^*Tie2cre mice, such that the BH_4_/BH_2_ and biopterin ratio was significantly reduced in *Gch1^fl/fl^*Tie2cre hearts (Fig. 1*L*). However, the BH_4_/BH_2_ and biopterin ratio in isolated endothelial cells was comparable between wild-type and *Gch1^fl/fl^*Tie2cre mice (Fig. 1*N*). In contrast to the observations in endothelial cells, BH_4_ levels in isolated cardiomyocytes were similar between wild-type and *Gch1^fl/fl^*Tie2cre mice, indicating that the reduction in overall heart tissue BH_4_ levels in *Gch1^fl/fl^*Tie2cre mice is due to specific deletion of endothelial cell *Gch1*. However, BH_2_ levels were significantly increased in cardiomyocytes isolated from *Gch1^fl/fl^*Tie2cre mice, such that the BH_4_/BH_2_ and biopterin ratio was significantly decreased in cardiomyocytes from *Gch1^fl/fl^*Tie2cre mice (Fig. 1, *O* and *P*), suggesting that selective endothelial cell BH_4_ deficiency in the heart leads to secondary effects on BH_4_ oxidation and/or recycling in cardiomyocytes, independent of changes in de novo BH_4_ biosynthesis. Importantly, plasma BH_4_ levels were similar between the groups, indicating that endothelial cell BH_4_ biosynthesis by GTPCH1 is not a major contributor to circulating BH_4_ levels (Supplemental Fig. S1: https://doi.org/10.6084/m9.figshare.21732581.v1).

### Endothelial Cell BH_4_ Deficiency Leads to Cardiac NOS Uncoupling with Increased Superoxide Production and Loss of Cardiac NO Generation

**Figure 2.**
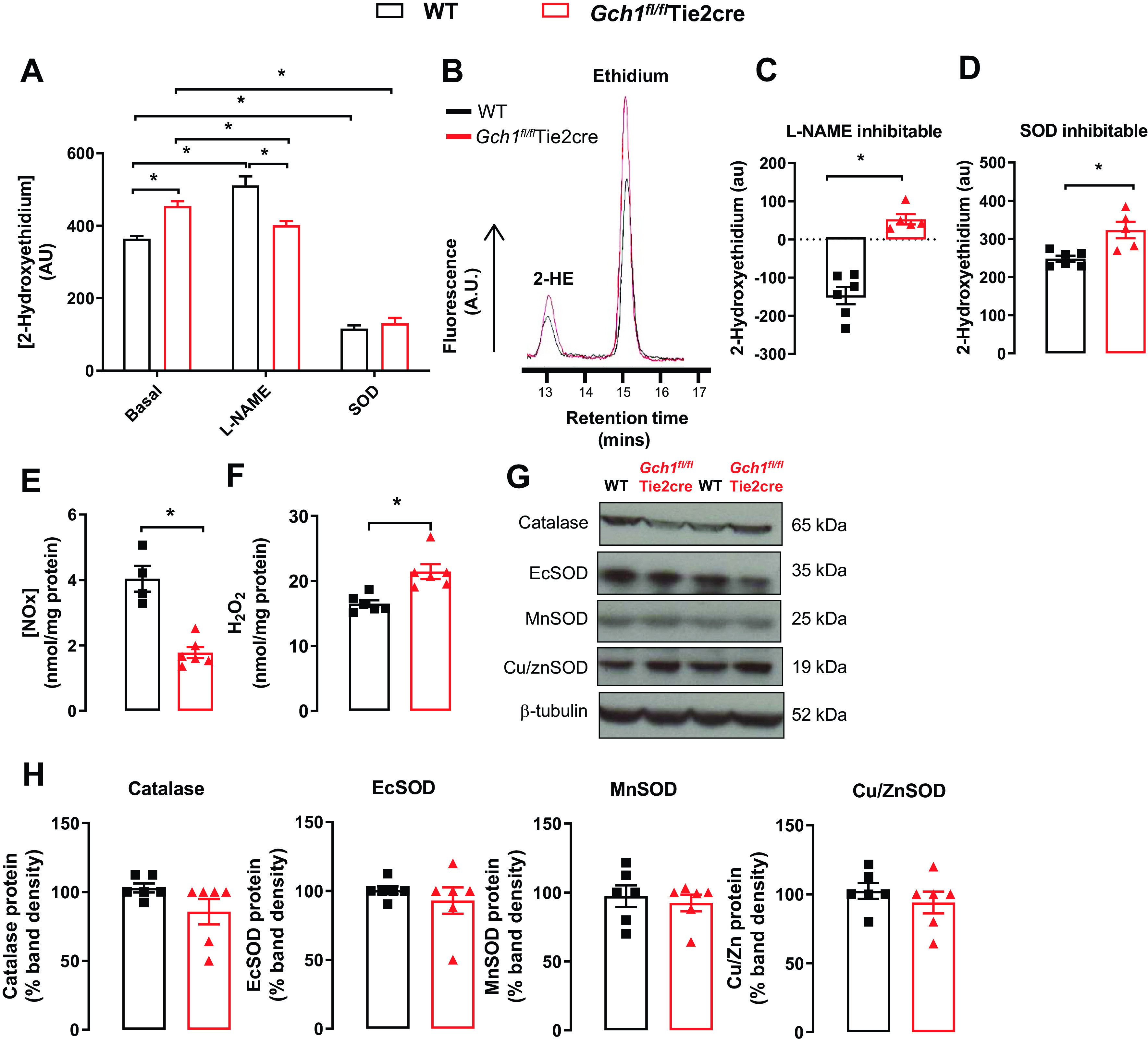
Superoxide production is increased, and nitric oxide bioavailability is reduced in cardiac endothelial cell BH_4_-deficient mice. Quantification of superoxide production, as measured by 2-hydroxyethidium (2-HE), in whole heart homogenate from *Gch1^fl/fl^*Tie2cre and wild-type (WT) mice using dihydroethidine (DHE) high-performance liquid chromatograph (HPLC). *A*: superoxide production was markedly increased in hearts from *Gch1^fl/fl^*Tie2cre mice compared with wild-type controls (*P < 0.05, *n* = 5–6 per group). *B*: representative trances of 2-HE and ethidium peaks in hearts from WT and *Gch1^fl/fl^*Tie2cre mice detected by DHE HPLC. *C* and *D*: nonselective nitric oxide synthase inhibitor *N*^G^-nitro-l-arginine methyl ester (l-NAME; 100 µM)-inhibitable fraction and polyethylene glycol superoxide dismutase, PEG-SOD (100 U/ml)-inhibitable fraction were greatly increased in *Gch1^fl/fl^*Tie2cre hearts compared with wild-type controls (*P < 0.05, *n* = 5–6 per group), respectively. *E*: nitrite/nitrate production in whole heart homogenate. Nitrite/nitrate production in heart homogenate from *Gch1^fl/fl^*Tie2cre mice was significantly decreased when compared with that from WT controls (*P < 0.05; *n* = 4–6 animals per group). *F*: levels of hydrogen peroxide from wild-type and *Gch1^fl/fl^*Tie2cre hearts were determined using an amperometric hydrogen peroxide microsensor electrode. Level of hydrogen peroxide production was significantly increased in endothelial cell BH_4_-deficient hearts (*Gch1^fl/fl^*Tie2cre) compared with wild-type controls (*P < 0.05; *n* = 6 per group). *G*: representative immunoblots for antioxidant proteins: catalase, Es-SOD, Mn-SOD, and Cu/Zn-SOD in *Gch1^fl/fl^*Tie2cre and wild-type hearts, with quantitative data, measured as percent band density in *H* (*n* = 6 per group). Each data point represents an individual adult male mouse.

We next determined the effects of altering cardiac endothelial cell BH_4_ availability on NOS function. We first measured basal superoxide productions in whole heart homogenate by quantification of 2-hydroxyethidium (2-HE) production from dihydroethidine, using high-performance liquid chromatography (HPLC). Basal superoxide production was significantly elevated in hearts from *Gch1^fl/fl^*Tie2cre mice compared with wild-type controls (*P* < 0.05, Fig. 2, *A* and *B*). In the presence of the nonselective nitric oxide synthase inhibitor l-NAME (100 µM), the levels of NOS-derived superoxide production in wild-type hearts were significantly increased compared with untreated hearts, suggesting a tonic scavenging effect of cardiac NO on superoxide. Furthermore, there was significant inhibition of superoxide production in *Gch1^fl/fl^*Tie2cre hearts by the NOS inhibitor l-NAME, (*P* < 0.05, Fig. 2, *A*–*D*), suggesting that NOS is a source of superoxide production in *Gch1^fl/fl^*Tie2cre endothelial cells.

To determine the effects of endothelial cell BH_4_ deficiency on NO bioactivity, we measured nitrate and nitrite in heart homogenates using ozone chemiluminescence. Nitrite and nitrate production were significantly reduced in *Gch1^fl/fl^*Tie2cre hearts compared with wild-type controls (*P* < 0.05, Fig. 2*E*). Furthermore, hydrogen peroxide production was significantly elevated in endothelial cell BH_4_-deficient hearts compared with wild-type controls (*P* < 0.05, Fig. 2*F*). To investigate whether increased productions of superoxide and hydrogen peroxide and reduced NO bioactivity in *Gch1^fl/fl^*Tie2cre heart altered antioxidant defenses, we measured protein levels of antioxidant enzymes by Western blot. There was no change in protein levels of catalase, extracellular superoxide dismutase (ecSOD), manganese superoxide dismutase (MnSOD), or Cu/ZnSOD between *Gch1^fl/fl^*Tie2cre and wild-type hearts (Fig. 2, *G* and *H*). Taken together, these data demonstrate that deficiency in coronary endothelial cell BH_4_ leads to eNOS uncoupling, increased superoxide and hydrogen peroxide productions, and decreased NO bioactivity in myocardium from *Gch1^fl/fl^*Tie2cre mice.

### Specific Loss of Endothelial Cell BH_4_ Leads to Cardiac Dysfunction and Hypertrophy

**Figure 3.**
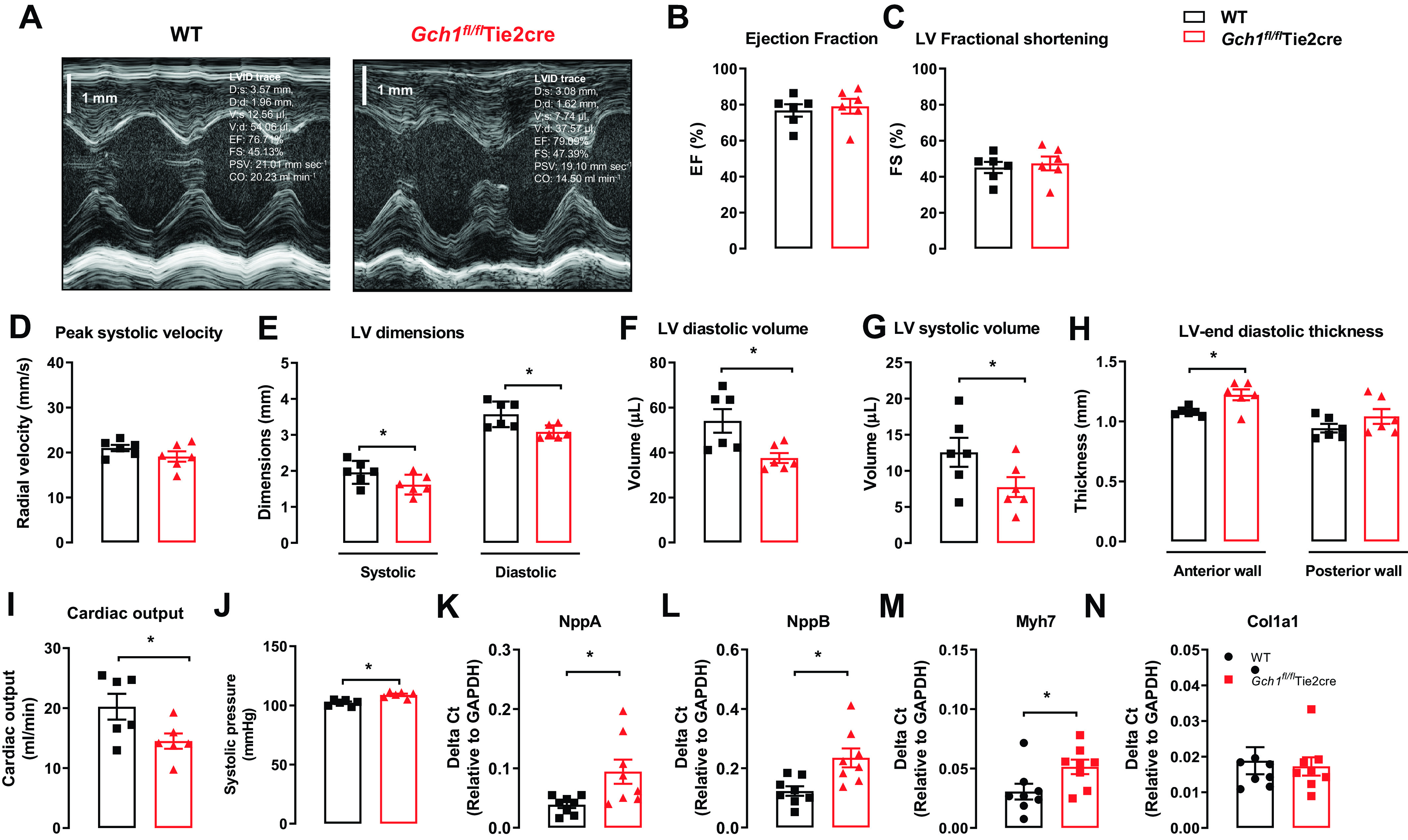
Loss of cardiac endothelial cell BH_4_ leads to cardiac dysfunction and hypertrophy. *A*: example of M-mode echocardiograms from *Gch1^fl/fl^*Tie2cre and wild-type littermates controls. *B*: ejection fraction. *C*: LV fractional shortening. *D*: peak systolic velocity, cardiac output (mL/min). *E*: systolic and diastolic LV end dimensions (mm). *F* and *G*: LV diastolic and systolic volume (μL). *H*: anterior and posterior LV end-diastolic thickness (mm). *I*: cardiac output (mL/min) in WT and *Gch1^fl/fl^*Tie2cre mice (*P < 0.05, *n* = 6 per group). *J*: systolic blood pressure in *Gch1^fl/fl^*Tie2cre and wild-type littermates were determined using tail-cuff plethysmography. *K–M*: gene expression of hypertrophic markers and fibrosis marker (*N*) in hearts from *Gch1^fl/fl^*Tie2cre and WT littermates (*P < 0.05*; n* = 7–8 per group). Each data point represents an individual adult male mouse.

We next investigated the effect of endothelial cell *Gch1* and BH_4_ deficiency on cardiac function, using M-mode echocardiography (Fig. 3*A*). There was no difference in either fractional shortening or ejection fraction or peak systolic velocity in wild-type and *Gch1^fl/fl^*Tie2cre mice (Fig. 3, *B*–*D*). However, left ventricular (LV) diastolic volume and LV end dimensions were significantly reduced in *Gch1^fl/fl^*Tie2cre mice compared with wild-type littermates (Fig. 3*E*). LV diastolic and systolic volume were also significantly reduced in *Gch1^fl/fl^*Tie2cre mice compared with wild-type littermates (Fig. 3, *F* and *G*). LV end-diastolic thickness was significantly increased in *Gch1^fl/fl^*Tie2cre mice compared with wild-type littermates (Fig. 3*H*). Cardiac output was significantly depressed in *Gch1^fl/fl^*Tie2cre mice compared with wild-type controls (Fig. 3*I*). We next measured blood pressure in *Gch1^fl/fl^*Tie2cre and wild-type littermate controls using tail-cuff plethysmography. We observed that *Gch1^fl/fl^*Tie2cre mice have a mild increased (∼5–7 mmHg) systolic blood pressure compared with wild-type littermate controls (102 ± 3 mmHg WT vs. 109 ± 2 mmHg in *Gch1^fl/fl^*Tie2cre mice; Fig. 3*J*).

In addition, we have undertaken further studies to examine the effect of endothelial cell BH_4_ deficiency on blood glucose levels and lipid profiles in *Gch1^fl/fl^*Tie2cre mice. First, we observed that blood glucose levels after 6 h of fasting were also comparable between wild-type and *Gch1^fl/fl^*Tie2cre mice (Supplemental Fig. S1). Lipid profiles including total cholesterol, triglycerides, LDL, and HDL were also comparable between the groups (Supplemental Fig. S2). These findings indicate that endothelial cell BH_4_ deficiency does not affect the metabolic or lipid profile in *Gch1^fl/fl^*Tie2cre mice.

To investigate the downstream effect of endothelial cell BH_4_ deficiency and NOS uncoupling on myocardial remodeling and hypertrophy, we measured mRNA expression of fetal genes in heart homogenates from *Gch1^fl/fl^*Tie2cre mice and their littermates. The mRNA expression of hypertrophic markers including natriuretic factor type A (*Nppa*), type B (*Nppb*), and β-myosin heavy chain (*Myh7*) were significantly increased in *Gch1^fl/fl^*Tie2cre hearts compared with littermate WT controls (Fig. 3, *K*–*M*). In contrast, there was no difference in *Col1a1* (a fibrosis marker) expression between the genotypes (Fig. 3*N*). Taken together, these findings suggest that endothelial cell BH_4_ deficiency leads to LV dysfunction and hypertrophy.

### Endothelial Cell BH_4_ Deficiency Leads to Activation of Cardiomyocytes, Increased nNOS, and Decreased eNOS Protein in *Gch1^fl/fl^*Tie2cre Hearts

**Figure 4.**
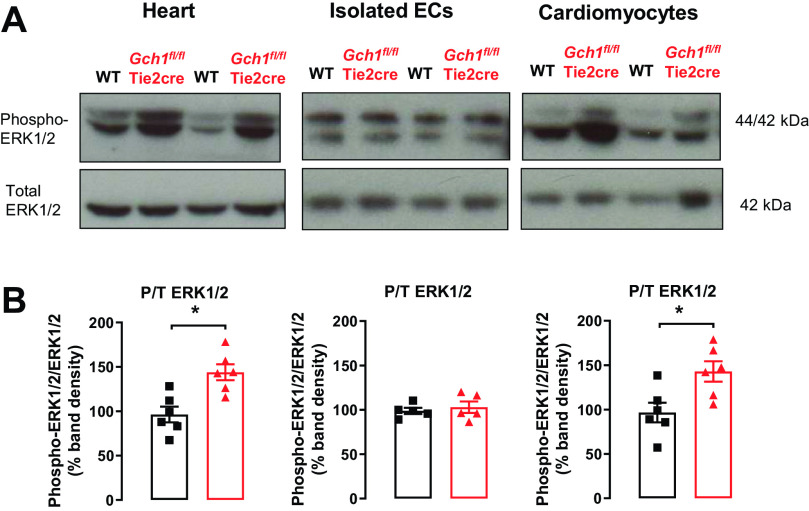
Increased phosphorylated extracellular signal-regulated kinases 1/2 in hearts and cardiomyocytes from *Gch1^fl/fl^*Tie2cre mice. *A*: representative immunoblots for phosphorylated and total proteins for extracellular signal-regulated kinases 1/2 (ERK1/2) in hearts, isolated endothelial cells (ECs), and cardiomyocytes from *Gch1^fl/fl^*Tie2cre mice and wild-type littermate controls. *B*: summary data (*P < 0.05, *n* = 5–6 per group). Each data point represents an individual adult male mouse.

To investigate the mechanism by which cardiac endothelial cell-specific BH_4_ deficiency impairs of cardiac function and increases hypertrophy, we isolated primary endothelial cells and primary cardiomyocytes from *Gch1^fl/fl^*Tie2cre and wild-type mice and tested whether NOS uncoupling from endothelial cells could mediate changes in cardiomyocytes. First, we observed that Erk1/2 phosphorylation was significantly increased in the whole hearts from *Gch1^fl/fl^*Tie2cre mice compared with wild-type littermate controls (Fig. 4, *A* and *B*). Furthermore, we found that Erk1/2 phosphorylation was significantly increased in primary cardiomyocytes from *Gch1^fl/fl^*Tie2cre compared with wild-type controls (Fig. 4, *A* and *B*). In contrast, we did not observe an increase in Erk1/2 phosphorylation in endothelial cells isolated from *Gch1^fl/fl^*Tie2cre hearts compared with littermate controls (Fig. 4, *A* and *B*). These findings suggest that loss of BH_4_ leads to NOS uncoupling in cardiac endothelial cells, which in turn causes changes in cardiomyocyte signaling pathways.

**Figure 5.**
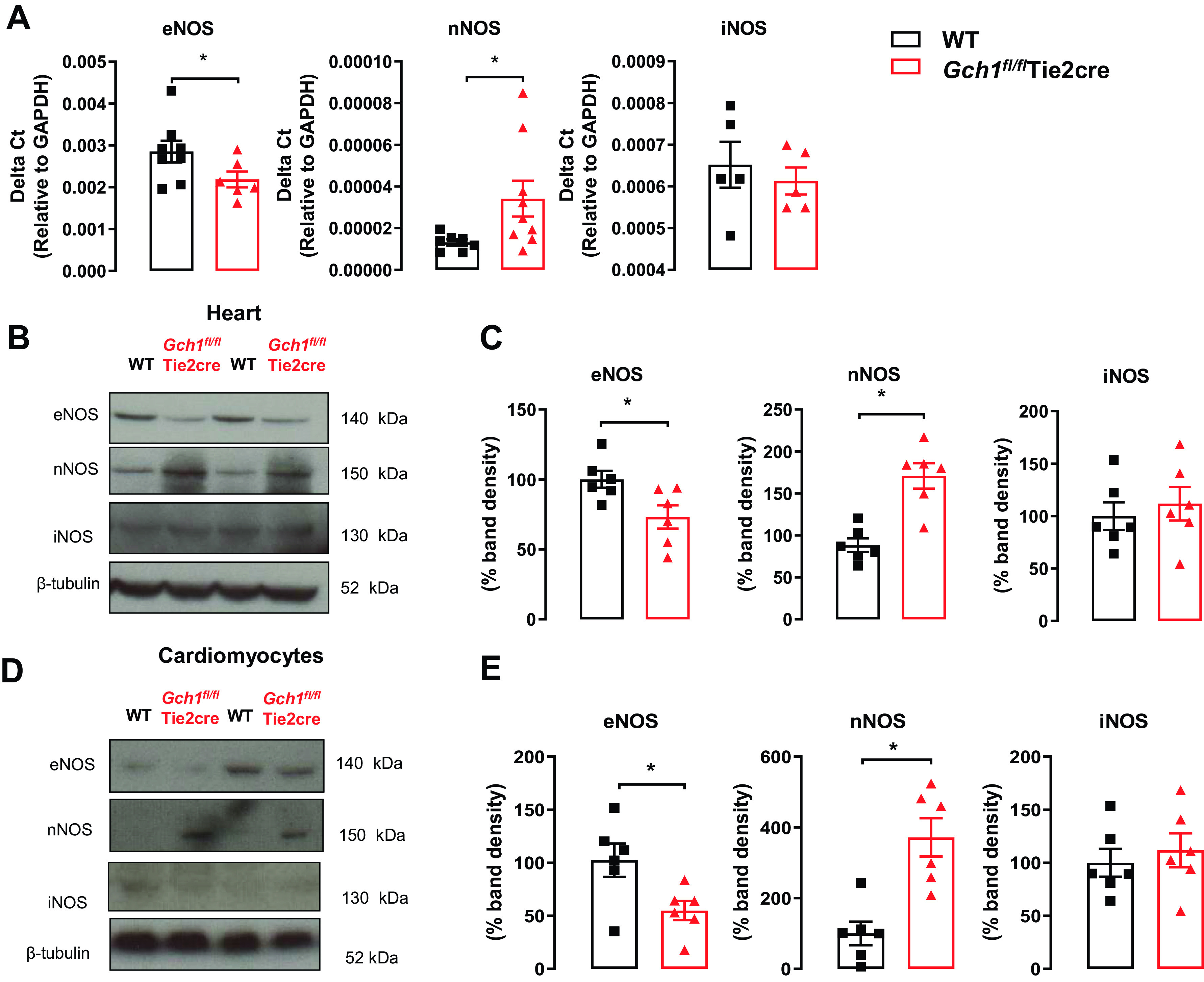
Deficiency in endothelial cell BH_4_ causes an increased nNOS expression and reduced eNOS expression in the hearts specifically in cardiomyocytes from *Gch1^fl/fl^*Tie2cre mice. *A*: quantitative real-time PCR was used to quantify endothelial cell nitric oxide synthase (eNOS), neuronal NOS (nNOS), and inducible NOS (iNOS) gene expression in hearts from *Gch1^fl/fl^*Tie2cre mice and wild-type controls (*P < 0.05; *n* = 5–9 per group). *B*: representative immunoblots of eNOS, nNOS, and iNOS isoforms in hearts from *Gch1^fl/fl^*Tie2cre mice and wild-type controls. *C*: summary data (*P < 0.05; *n* = 6 per group). *D*: representative immunoblots of eNOS, nNOS, and iNOS isoforms in cardiomyocytes from *Gch1^fl/fl^*Tie2cre mice and wild-type controls. *E*: summary data (*P < 0.05*; n* = 6 per group). Each data point represents an individual adult male mouse.

We next determined whether cardiac dysfunction and hypertrophy in *Gch1^fl/fl^*Tie2cre hearts are associated with changes in myocardial NOS isoforms. Quantitative real-time PCR and Western blot analysis demonstrated a significant increase in nNOS mRNA and protein in *Gch1^fl/fl^*Tie2cre hearts, accompanied by a reduction in eNOS mRNA and protein (Fig. 5, *A*–*C*). There was no significant difference in iNOS mRNA and protein expression between *Gch1^fl/fl^*Tie2cre hearts and wild-type hearts (Fig. 5, *A*–*C*). To investigate which cell type is responsible for the upregulation of nNOS and downregulation of eNOS protein, NOS isoforms were determined in isolated cardiomyocytes from *Gch1^fl/fl^*Tie2cre mice, revealing increased nNOS protein and decreased eNOS protein in isolated cardiomyocytes from *Gch1^fl/fl^*Tie2cre hearts (Fig. 5, *D* and *E*).

**Figure 6.**
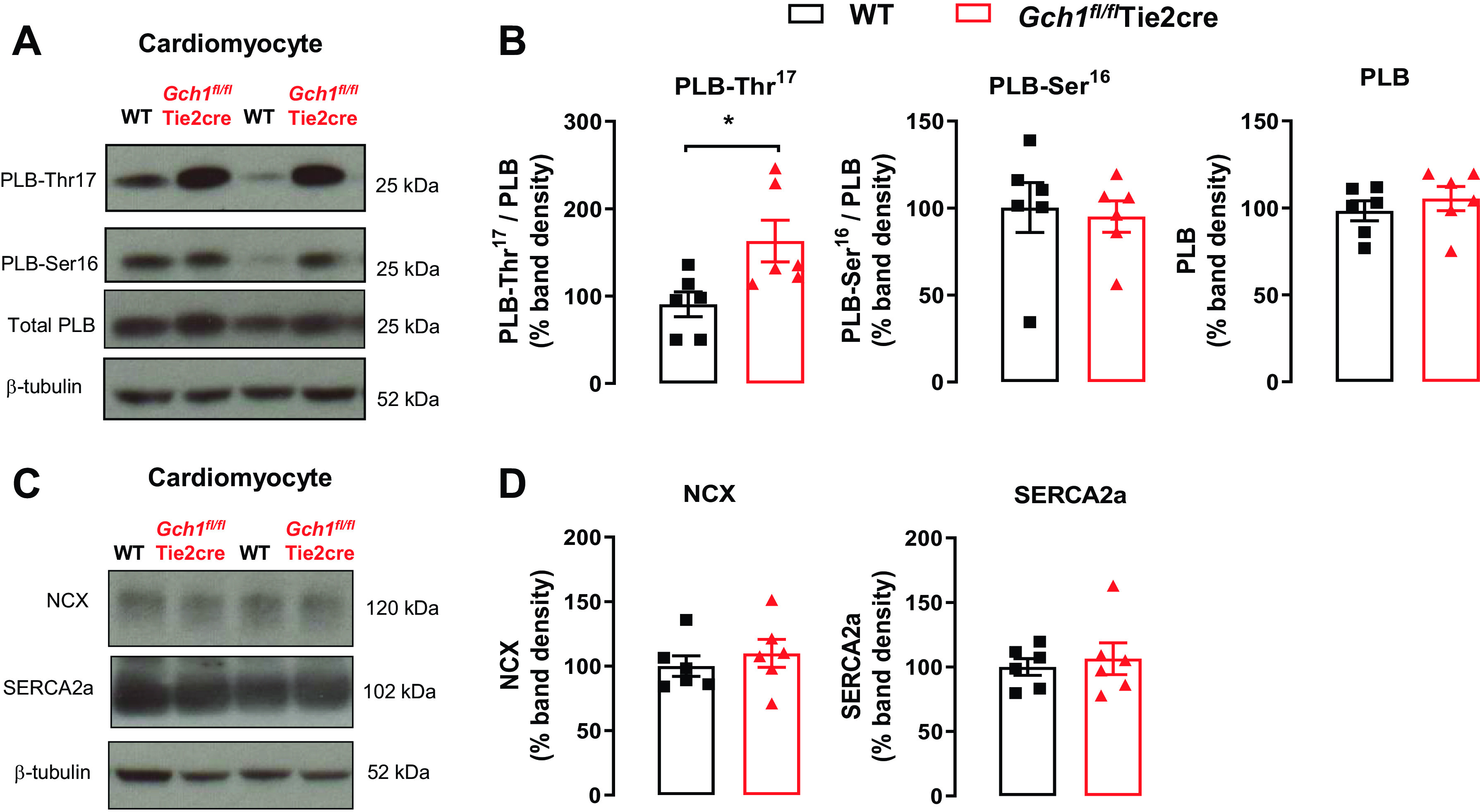
Calcium-handling proteins in cardiomyocytes from wild-type and *Gch1^fl/fl^*Tie2cre mice. *A* and *B*: phosphorylation of phospholamban (PLB) at Thr (17) was significantly increased in cardiomyocytes from *Gch1^fl/fl^*Tie2cre mice (*P < 0.05; *n* = 6 per group), whereas the phosphorylation of phospholamban at Ser (16) was unchanged (*n* = 6 per group). *C*: representative immunoblots for sodium-calcium exchanger (NCX) and sarco(endo)plasmic reticulum Ca^2+^ ATPase (SERCA) in cardiomyocytes from *Gch1^fl/fl^*Tie2cre mice and wild-type littermate controls. *D*: summary data (*n* = 6 per group). Each data point represents an individual adult male mouse.

We further investigated whether loss of *Gch1*/BH_4_ in endothelial cells alters calcium-handling proteins in cardiomyocytes. We found that phospholamban phosphorylation at the calmodulin-dependent kinase II (CaMKII)-specific site (PLB-Thr17) was increased in myocardium from endothelial cell BH_4_-deficient mice, but not at the protein kinase A-specific site (PLB-Ser16) (Fig. 6, *A* and *B*). There was no change in overall protein levels of phospholamban, NCX, or SERCA2A in cardiomyocytes between *Gch1^fl/fl^*Tie2cre and wild-type mice (Fig. 6, *C* and *D*). Taken together, these data demonstrate that endothelial cell BH_4_ deficiency leads to activation of cardiomyocytes, increased nNOS, and decreased eNOS protein in *Gch1^fl/fl^*Tie2cre hearts, associated with alteration in cardiomyocyte calcium handling.

### Deficient Endothelial Cell *Gch1*/BH_4_ Biosynthesis Leads to Coronary Vascular Dysfunction and Injury Following Cardiac Ischemia-Reperfusion

**Figure 7.**
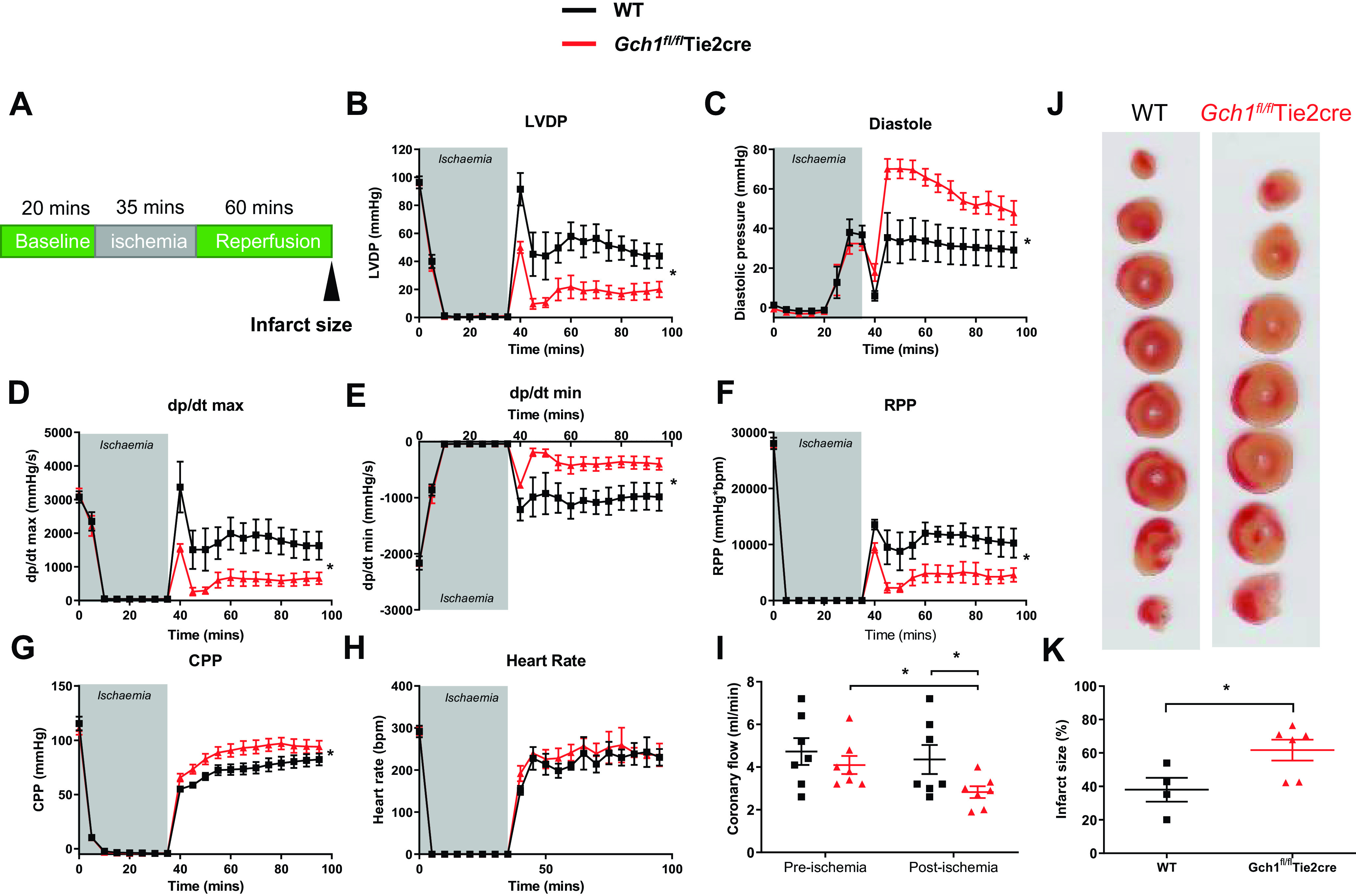
Loss of endothelial cell *Gch1*/BH_4_ biosynthesis leads to cardiac dysfunction and injury following cardiac ischemia-reperfusion. Cardiac function and infarct size were measured in isolated wild-type (WT) and *Gch1^fl/fl^*Tie2cre hearts perfused using Langendorff model. *A*: after baseline stabilization, wild-type and *Gch1^fl/fl^*Tie2cre hearts were subjected to 35 min of global ischemia and 60 min of reperfusion. *B*: LV developed pressure (LVDP). *C*: LV diastolic pressure. *D*: LV dP/d*t*_max_. *E*: LV dP/d*t*_min_. *F*: rate pressure product (RPP; mmHg). *G*: coronary perfusion pressure (CPP; mmHg). *H*: heart rate (beats/min). *I*: coronary flow was measured in hearts before ischemia and after periods of ischemia followed by 60 min of reperfusion (*n* = 7 per group). *J* and *K*: infarct size, defined by triphenyltetrazolium chloride staining and measurement as percentage of risk region, was significantly increased in hearts from *Gch1^fl/fl^*Tie2cre mice compared with wild-type littermate controls. Values are the means ± SE (*P < 0.05; *n* = 4–6 animals per group). Each data point represents an individual adult male mouse.

To investigate the specific role of endothelial cell BH_4_ in postischemic myocardial function and injury following ischemia-reperfusion, Langendorff perfused wild-type and *Gch1^fl/fl^*Tie2cre hearts were subjected to 35 min global ischemia followed by 60-min reperfusion (Fig. 7*A*). Myocardial function was measured using a ventricular balloon to determine LV developed pressure, LV end-diastolic pressure, and rate pressure product. LV functional recovery after 35 min of global ischemia was significantly impaired in *Gch1^fl/fl^*Tie2cre hearts compared with wild-type controls. This impairment in recovery was manifest as decreased LV-developed pressure (Fig. 7*B*) and increased diastolic pressure (Fig. 7*C*). The maximal rates of contraction (LV dP/d*t*_max_) and relaxation (LV dP/d*t*_min_) were also significantly lower in the *Gch1^fl/fl^*Tie2cre hearts than in the wild-type group (*P* < 0.05) (Fig. 7, *D* and *E*). The rate pressure product (RPP), an index of workload, was significantly decreased in *Gch1^fl/fl^*Tie2cre hearts compared with wild-type controls (Fig. 7*F*). The coronary perfusion pressure (CPP), a direct measure of coronary vascular resistance, was greatly increased in *Gch1^fl/fl^*Tie2cre hearts compared with wild-type controls (Fig. 7*G*). Importantly, the coronary flow was significantly reduced after ischemia in *Gch1^fl/fl^*Tie2cre hearts compared with wild-type hearts (Fig. 7*I*). In addition, in *Gch1^fl/fl^*Tie2cre hearts, infarct size, assessed by TTC staining, was significantly larger than in wild-type mice (*Gch1^fl/fl^*Tie2cre, 62 ± 6% of the risk region; wild type, 38 ± 7%, *P* < 0.05; Fig. 7, *J* and *K*). Collectively, these data demonstrate a critical role for endothelial cell *Gch1*/BH_4_ biosynthesis on coronary vascular function, cardiac function, and myocardial injury following ischemia-reperfusion injury.

## DISCUSSION

In this study, we used a mouse model of endothelial cell-targeted *Gch1* deletion to test the specific requirement for endothelial cell BH_4_ in the regulation of cardiac function under both physiological and pathophysiological conditions. The major findings of this study are as follows: *1*) endothelial cell-targeted *Gch1* deletion leads to selective loss of endothelial cell BH_4_ in the myocardium; *2*) loss of endothelial cell BH_4_ leads to NOS uncoupling, increased superoxide and hydrogen peroxide productions, and decreased NO bioactivity, resulting in cardiac dysfunction and mild myocardial hypertrophy; *3*) selective loss of endothelial cell BH_4_ is associated with changes in cardiomyocytes, including increased nNOS protein, loss of eNOS protein, and increased in phospholamban phosphorylation at Ser17; and *4*) specific loss of cardiac endothelial cell BH_4_ leads to coronary vascular dysfunction, cardiac dysfunction, and increased myocardial infarct size following ischemia-reperfusion injury. Collectively, these studies reveal specific effects of endothelial cell *Gch1*/BH_4_ biosynthesis on cardiomyocytes and on overall cardiac function under both physiological and pathophysiological conditions.

An important observation underpinning the interpretation of selective and specific roles for endothelial cell BH_4_ is that endothelial cell-targeted *Gch1* deletion abolishes GTPCH protein expression and de novo BH_4_ biosynthesis in endothelial cells, and this loss of endothelial cell BH_4_ synthesis is not rescued by normal levels of BH_4_ in plasma or adjacent cells. This indicates that BH_4_ in endothelial cells is compartmentalized and is not amenable to uptake or recycling, at least under conditions of normal BH_4_ levels. High-level supplementation of sepiapterin (20, 21, 33) or BH_4_ (34, 35) has been shown to prevent NOS uncoupling and improved left ventricular function and I/R injury. However, it is not clear whether these effects of supraphysiological BH_4_ supplementation are mediated by known BH_4_ functions, and if so via which cell type and which mechanisms. Previous observations suggest that exogenous BH_4_ is not sufficient to rescue or augment BH_4_ levels in endothelial cells (30), whereas cardiomyocytes are amenable to exogenous BH_4_ supplementation (18, 23, 36).

Our study emphasizes the importance of the cross talk between the coronary endothelium and cardiomyocytes in cardiac function (37). We found that selective loss of endothelial cell BH_4_ led to changes in cardiomyocyte redox signaling, gene expression, and function. Increased superoxide and hydrogen peroxide generation in endothelial cell BH_4_-deficient hearts is consistent with the induction of fetal gene expression (*nppa*, *nppb*, and *myh7*) contributing to cardiac hypertrophy. Indeed, emerging evidence suggests that H_2_O_2_ can mediate and cause cardiac dysfunction, hypertrophy, and heart failure (38–40). In eNOS knockout mice, cardiac structure, LV function, and heart rate were similar to that of wild-type mice (41). These findings suggest that the combination of increased NOS-derived H_2_O_2_ and reduced NOS-derived NO production are likely to be important contributors to the development of cardiac dysfunction in *Gch1^fl/fl^*Tie2cre mice and contrasts with eNOS knockout mice where all functions of eNOS (i.e., both NO and ROS generation) are deleted.

Neuronal NOS (nNOS) has also been implicated in the regulation of basal and β-adrenergic inotropy in normal and chronically infarcted hearts. We confirmed that nNOS was upregulated and eNOS downregulated in cardiomyocytes in endothelial cell BH_4_-deficient mice. Consistent with these findings, increased nNOS and decreased eNOS expression have been observed in the failing rat and human hearts (42–44). It is possible that upregulation of eNOS-derived H_2_O_2_ generation from coronary endothelial cells from *Gch1^fl/fl^*Tie2cre mice affects eNOS protein and activity in the cardiomyocytes. Consistent with this idea, several reports have shown that H_2_O_2_ decreases eNOS protein expression and activity, at least in part by an inhibition of c-Jun activity and thus leading to a reduction in AP-1 transcription factor binding to the eNOS promoter (45–47). Thus, eNOS expression and activity have been found to be suppressed in the failing hearts, suggesting that the eNOS-mediated regulation of cardiac function and β-adrenergic response may be reduced in myocardial disease.

We have shown that *Gch1^fl/fl^*Tie2cre mice have a slightly increased (∼5–7 mmHg) in systolic blood pressure compared with wild-type littermate controls. Thus, it is possible that increased systolic blood pressure in *Gch1^fl/fl^*Tie2cre mice may contribute to cardiac dysfunction and hypertrophy in this study. However, this degree of blood pressure elevation is very modest and would not usually be considered sufficient to constitute a model of “hypertension.” Indeed, the eNOS knockout mouse has systemic hypertension with a 20–30-mmHg increase in systolic blood pressure, but at baseline, cardiac contractility was reported to be normal in eNOS knockout mice (48, 49). Therefore, it is unlikely that a mild increase in systolic blood pressure in *Gch1^fl/fl^*Tie2cre mice was responsible for the cardiac dysfunction and hypertrophy in this study.

Increased superoxide and hydrogen peroxide productions in the myocardium have been shown to impair calcium handling and induce pathological cardiac changes such as fibrosis, apoptosis, and hypertrophy (50–52). We found that endothelial cell BH_4_-deficient mice had increased phospholamban phosphorylation at the calmodulin-dependent kinase II (CaMKII)-specific site (PLB-Thr17) but not at the protein kinase A-specific site (PLB-Ser16). nNOS modulates cardiac relaxation via effects on phospholamban phosphorylation (53). PLB has inhibitory effect on SERCA activity and reuptake of Ca^2+^. Therefore, increased phospholamban phosphorylation at Ser17 abrogates the inhibitory effect of PLB on SERCA, thereby increasing SR Ca^2+^ reuptake and ultimately increased myocyte contractility and relaxation. This finding suggests that increased nNOS gene expression and protein in *Gch1^fl/fl^*Tie2cre hearts and cardiomyocytes may contribute to an increase in contraction and accelerated SR Ca^2+^ reuptake in cardiomyocytes, possibly by increased basal PLB phosphorylation.

Myocardial ischemia-reperfusion is associated with markedly elevated levels of ROS production (14, 54), and these ROS are central mediators of postischemic injury. In the postischemic heart, changes in coronary endothelial vascular function occur because of a reduction in eNOS-derived NO production which in turn impairs coronary flow (24). Evidence from isolated rat hearts suggests that ischemia-induced oxidative stress leads to enhanced BH_4_ oxidation (11, 24), contributing to postischemic eNOS uncoupling with a resultant loss of coronary endothelium-dependent vasodilation (24). Our observation of greatly increased coronary perfusion pressure (CPP), a direct measure of coronary vascular resistance, and reduced coronary flow in *Gch1^fl/fl^*Tie2cre hearts now demonstrates a specific role of endothelial cell BH_4_ in determining the response of the coronary microcirculation to ischemia-reperfusion. Importantly, we found that loss of endothelial cell BH_4_ caused a greater myocardial infarct size compared with wild-type hearts following I/R.

Collectively, these data demonstrate a critical role for endothelial cell *Gch1*/BH_4_ biosynthesis in coronary vascular function, cardiac function, and the response to ischemia-reperfusion injury. Thus, targeting endothelial cell *Gch1* and BH_4_ biosynthesis may provide a novel therapeutic target for the prevention and treatment of cardiac dysfunction, ischemia injury, and heart failure.

## DATA AVAILABILITY

Data will be made available upon reasonable request.

## SUPPLEMENTAL DATA

10.6084/m9.figshare.21732581.v1 Supplemental Figs. S1 and S2: https://doi.org/10.6084/m9.figshare.21732581.v1.

## GRANTS

This study was supported by British Heart Foundation (BHF) Programme Grants RG/12/5/29576 and RG/17/10/32859, BHF Chair Award CH/16/1/32013, Wellcome Trust Grant 090532/Z/09/Z, BHF Centre of Research Excellence (Oxford) Grants RE/13/1/30181 and RE/18/3/34214, and the National Institute for Health Research (NIHR) Oxford Biomedical Research Centre.

## DISCLOSURES

No conflicts of interest, financial or otherwise, are declared by the authors.

## AUTHOR CONTRIBUTIONS

S.C. and K.M.C. conceived and designed research; S.C., S.M.C., R.C., M.K., J.K.B., J.N.S., G.D., and M.J.C. performed experiments; S.C., S.M.C., R.C., M.K., J.K.B., J.N.S., G.D., and M.J.C. analyzed data; S.C., B.C., and K.M.C. interpreted results of experiments; S.C. prepared figures; S.C. and K.M.C. drafted manuscript; S.C. and K.M.C. edited and revised manuscript; S.C., S.M.C., R.C., M.K., J.K.B., J.N.S., G.D., M.J.C., B.C., and K.M.C. approved final version of manuscript.
